# Clustering of the T-cell receptor complex may facilitate T-cell activation by stretching of CD3*ζ* cytoplasmic tails

**DOI:** 10.1007/s00249-026-01861-4

**Published:** 2026-07-27

**Authors:** Steven Siegl, Sebastian Fürthauer, Gerhard J. Schütz

**Affiliations:** Institute of Applied Physics, TU Wien, Lehargasse 6, 1060 Vienna, Austria

**Keywords:** T-cell biophysics, T-cell receptor, Signaling, Polymer physics, Transmembrane proteins, Intrinsically disordered proteins

## Abstract

Motivated by experimental observations that T-cells can activate as a consequence of clustering of T-cell receptors, we hypothesize that steric confinement between the intracellular chains of the TCR could play a role. Using coarse-grained simulations, we show that steric interactions between TCRs are sufficient to induce appreciable stretch of the intracellular domains of CD3*ζ* chains. We propose a model, in which the difference in membrane distances between the phosphatase CD45 and the kinases lck and fyn is able to sense the clustering-induced CD3*ζ* stretch: we estimate a 1.5- to 2-fold increase in phosphorylation of immunoreceptor tyrosine-based activation motifs (ITAMs) at CD3*ζ* upon TCR clustering.

**Supplementary Information:**

The online version contains supplementary material available at https://doi.org/10.1007/s00249-026-01861-4.

## Introduction

A remarkable feature of the vertebrate immune system is its inherent ability to distinguish harmful from harmless based on the primary antigen structure. T-cells embody this trait of adaptive immunity through their unique detection of antigens, which are displayed as peptide fragments in the context of the products of the major histocompatibility complex (MHC) on the surface of antigen-presenting cells (APCs). The formation of a productive immunological synapse, the area of contact between a T-cell and an APC (Grakoui et al. 1999), is driven by T-cell antigen receptors (TCRs) on the T-cell binding to peptide/MHC complexes (pMHC) on the APC.

TCRs are octameric complexes consisting in most cases of a highly variable $\alpha \beta$ heterodimer, which is noncovalently associated with the invariant CD3 dimers CD3$\epsilon \gamma$ and CD3$\epsilon \delta$ (heterodimers) and CD3$\zeta \zeta$ (homodimer). Upon pMHC binding to the $\alpha \beta$ heterodimer, phosphorylation of immunoreceptor tyrosine-based activation motifs (ITAMs) on the CD3 chains (primarily on CD3*ζ*) by the src-family kinase lck or fyn (Gascoigne et al. 2011) initiates the downstream T-cell signaling cascade. In turn, dephosphorylation via the phosphatase CD45 usually reverts ITAM phosphorylation (Van Der Merwe and Dushek 2011), keeping the net phosphorylation rate low under non-activating conditions. Shifting the balance between phosphorylation and dephosphorylation is hence generally considered highly relevant for the initiation of downstream signaling, and is commonly attributed to size-based exclusion of CD45’s large exoplasmic domain in the course of close contact formation upon antigen-binding (Davis and Van Der Merwe 2006). However, the molecular, biophysical and cellular mechanisms which underlie proximal TCR signal transduction remain opaque. In this manuscript, we explore the possibility of proximity-induced conformational changes of CD3*ζ* as a candidate for this.

Experimentally, there is evidence for a crucial role of CD3*ζ* configuration in antigen recognition (Aivazian and Stern 2000; Ma et al. 2020; Zhang et al. 2011): i) In the resting state, CD3*ζ* associates with the inner leaflet of the plasma membrane via acidic lipids (Aivazian and Stern 2000), where it assumes a partially *α*-helical conformation (Zimmermann et al. 2017). In this membrane-associated configuration CD3*ζ* is incompetent to ITAM-phosphorylation (Aivazian and Stern 2000), establishing membrane association as the default signaling-off state. ii) TCR engagement leads to dissociation of CD3*ζ* from the cytoplasmic leaf of the plasma membrane (Zhang et al. 2011), rendering the ITAMs accessible to the kinases lck and fyn and thereby switching CD3*ζ* to a phosphorylation-competent state. iii) Our own data indicate that artificial clustering of the TCR is sufficient to convert the receptor to a signaling-competent state (Hellmeier et al. 2021), and it has been shown that clustering of CD3*ζ* alone suffices to initiate T-cell triggering (Ma et al. 2020). Together, (i–iii) establish a mechanistic chain: membrane association sequesters ITAMs in the signaling-off state; dissociation from the membrane, whether triggered by antigen engagement or by steric crowding upon clustering, renders them phosphorylatable. Here we explore the clustering-driven arm of this mechanism in simulations.

CD3*ζ* is a single-pass transmembrane protein, which carries a short N-terminal extracellular segment connected to an *α*-helical transmembrane region, and a long, unstructured cytoplasmic region of 110 residues. Like the CD3$\epsilon \gamma$ and the CD3$\epsilon \delta$ heterodimers, the CD3$\zeta \zeta$ homodimer is disulfide-linked via cysteines close to the transmembrane region (Wucherpfennig et al. 2010). Each CD3*ζ* protomer contains 3 ITAMs, which together with single ITAMs per CD3*γ*, *δ*, and *ε*, add up to 10 ITAMs per TCR (Love and Hayes 2010). ITAMs themselves contain 2 tyrosine residues, the phosphorylation of which represents one of the earliest events following TCR engagement. Double phosphorylated ITAMs recruit the kinase ZAP-70 to the TCR, where it is activated and phosphorylates downstream substrates (Wang et al. 2010). It is hence not only relevant for the T-cell's response, *whether* ITAMs are phosphorylated, but also *to which degree* phosphorylation takes place (Love and Hayes 2010).

Using cryo-electron microscopy (cryo-EM) the structure of the TCR-CD3 complex was recently identified (Dong et al. 2019; Sušac et al. 2022), yielding the mutual arrangements of the transmembrane and exoplasmic domains in unprecedented detail: The two transmembrane helices of the CD3$\zeta \zeta$ homodimer form a coiled coil (Call et al. 2006) and further bind to basic residues of the TCR$\alpha \beta$ chains. In the cryo-EM images, however, the cytosolic parts of CD3*ζ* (CD3$\zeta _\text {cyt}$) did not reveal any electron density, indicating high conformational heterogeneity. This agrees with neutron reflection data, where two membrane-associated states of CD3$\zeta _\text {cyt}$ were detected – one deeply inserted and one peripherally bound –, which likely are in dynamic equilibrium (Zimmermann et al. 2017). Membrane association was critically dependent on the presence of acidic phospholipids, suggesting electrostatic interactions with the basic amino acids of CD3$\zeta _\text {cyt}$ to be responsible for its membrane association. Besides CD3*ζ* also CD3*ε* was reported to show membrane association, whereas CD3*γ* and CD3*δ* did not (Sigalov et al. 2006).

Literature about conformational changes of CD3*ζ* is scarce. In a pioneering study, van der Merwe et al. used Förster Resonance Energy Transfer (FRET) between fluorescent CD3*ζ* and a plasma membrane-inserted hydrophobic dye (Zhang et al. 2011). Indeed, decreased FRET was observed upon TCR triggering via beads coated with anti-CD3 antibodies. In addition, membrane-association was found to depend on the presence of basic-residues in CD3$\zeta _\text {cyt}$. Similar results were obtained in a more recent study by Huang and colleagues (Sasmal et al. 2020). In turn, artificial clustering of CD3*ζ* was found to be sufficient to stimulate ITAM phosphorylation (Ma et al. 2020; Spencer et al. 1993; Letourneur and Klausner 1991).

Based on these findings, we asked if the clustering of CD3$\zeta _\text {cyt}$ could facilitate T-cell activation by promoting the dissociation of ITAMs from the inner leaflet of the plasma membrane through steric interactions. A known effect in polymer brush systems, where multiple polymers are fixed at one end to a surface, is that the chains are forced to stretch along the normal direction of the surface if the polymer density is high enough (Zhao and Brittain 2000). In this study, our objective is to determine whether this effect occurs when clustering multiple CD3$\zeta _\text {cyt}$, even though the density of the polymer is relatively low.

To test this hypothesis, we performed coarse-grained molecular dynamics simulations comparing single CD3$\zeta _\text {cyt}$ chains with clustered configurations containing multiple CD3$\zeta _\text {cyt}$ chains. We show that steric interactions within a cluster enhance the likelihood and duration of ITAM dissociation events. We speculate that this potentially facilitates phosphorylation and signal transduction.

**Fig. 1:**
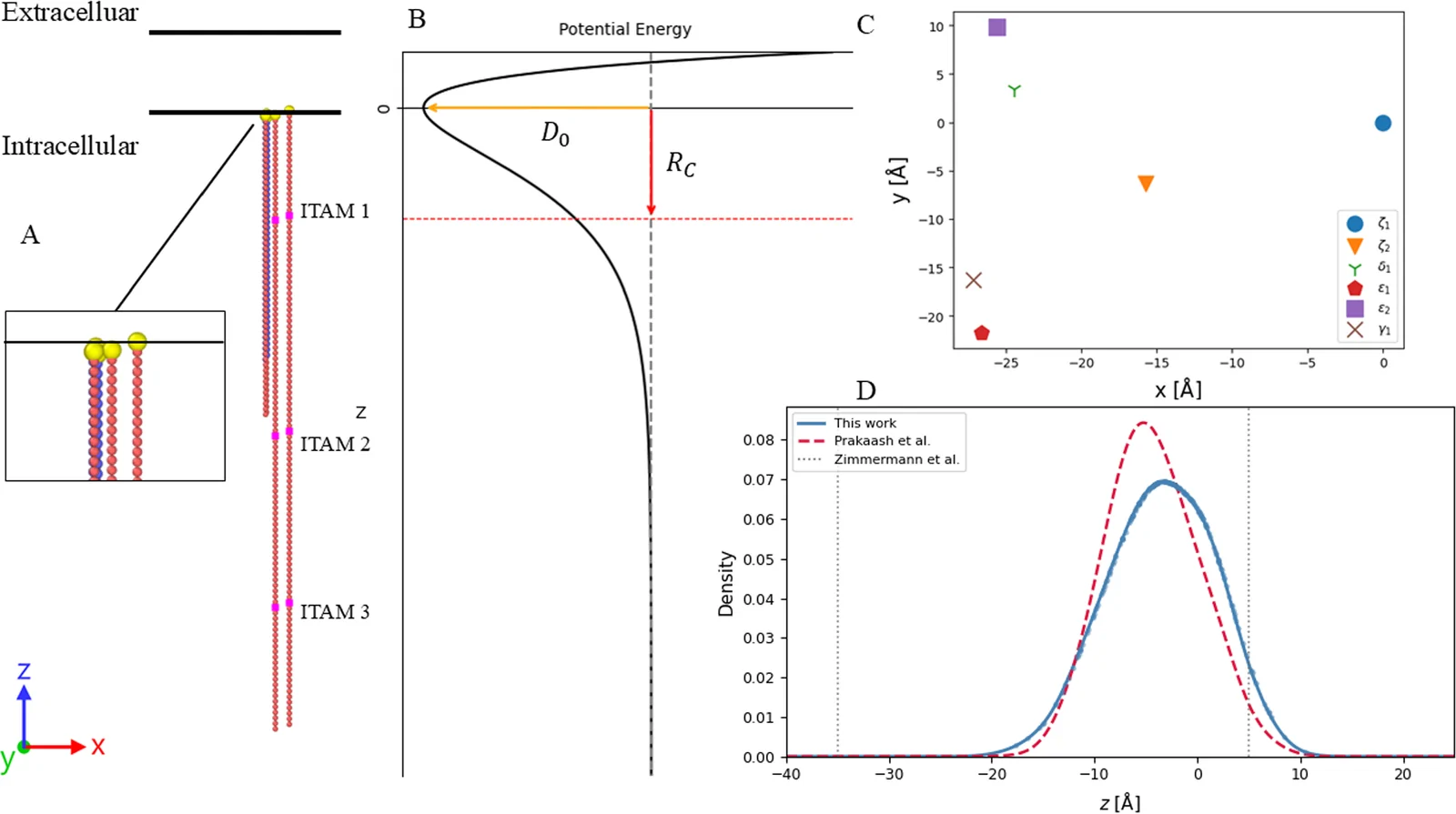
Visualization of the starting configuration and the membrane interaction profile of the 1-complex configuration. (**A**) Initial positions of the polymer chains in the 1-complex configuration. All six cytoplasmic chains are shown; two chains appear overlapping due to the viewing angle. Yellow units represent the link to the transmembrane domains and are hence fixed throughout the simulation. Red units correspond to CD3*ζ* (long strands) and CD3*ε* and interact with the Morse potential, while blue units corresponding to CD3*γ* and CD3*δ* do not show membrane affinity and hence do not interact with the Morse potential. Pink units indicate the position of the first tyrosine residue in each ITAM. (**B**) Morse potential used to model membrane interactions. The dotted line marks the cutoff distance beyond which the potential is set to zero. (**C**) Fixed relative positions of the first unit in each chain for the 1-complex configuration. (**D**) Distribution of the mean *z*-position of the eight membrane-proximal $\zeta _1$ beads in our simulation (blue curve, this work) compared to the $\zeta _1$ cytoplasmic region center of mass extracted from the coarse-grained trajectories of Prakaash et al. (Prakaash et al. 2021) (red dashed curve). In this and all subsequent figures, *z = 0* corresponds to the inner leaflet of the plasma membrane; negative *z* values denote the cytoplasmic direction. Both distributions peak at $z \approx -4\,$ Å, reflecting membrane association of CD3$\zeta _\text {cyt}$. The gray dotted lines indicate the range of the $\zeta _\text {CYT}$ occupied volume reported by Zimmermann et al. (Zimmermann et al. 2017); their stated peak of *15 * Å is an absolute distance from the membrane surface, corresponding to $z \approx -15\,$ Å in our convention

## Methods

We performed coarse-grained simulations, using LAMMPS (Thompson et al. 2022), of all cytoplasmic CD3 domains of a TCR complex, representing each of the six constituent proteins as independent polymer chains, with one unit per amino acid. The number of residues per chain was determined from PDB entries (Saotome et al. 2023), focusing exclusively on the cytoplasmic regions.

Membrane interactions were modeled using a Morse potential for the *ζ* and *ε* chains, reflecting their reported membrane affinity (Wucherpfennig et al. 2010; Xu et al. 2008). The *γ* and *δ* chains, which lack such interactions, were confined using a harmonic wall potential ($\epsilon = 10\,k_BT/$ Å^2^, cutoff *50 * Å above the inner leaflet) to prevent unphysical escape into the extracellular space. The spring constant was chosen to be large enough to keep the chains cytoplasmic while remaining sufficiently soft to avoid significantly perturbing their configurational statistics.

Each amino acid is represented by a single coarse-grained bead; all beads are of the same generic type, i.e. bead identity does not encode amino-acid chemistry. Bonded interactions between neighbouring beads along the chain are described by a harmonic bond potential (spring constant $k = 100\,k_BT/$Å^2^, equilibrium length *6.4 * Å, corresponding to the C*α*–C*α* distance per residue). Non-bonded excluded-volume interactions between all pairs of beads are modelled with a Lennard-Jones potential ($\epsilon _\text {LJ} = 0.3\,k_BT$, $\sigma = 5.73\,$ Å, cutoff *6.43 * Å, shifted to zero at the cutoff), giving a purely repulsive interaction at physiological conditions.

The first unit of each chain was fixed in space. The positions of the first unit were based on cryo-electron microscopy structural data (Saotome et al. 2023), with the coordinate origin defined in the first unit of the $\zeta _1$ chain. These positions are shown in fig. 1(C).

**Fig. 2:**
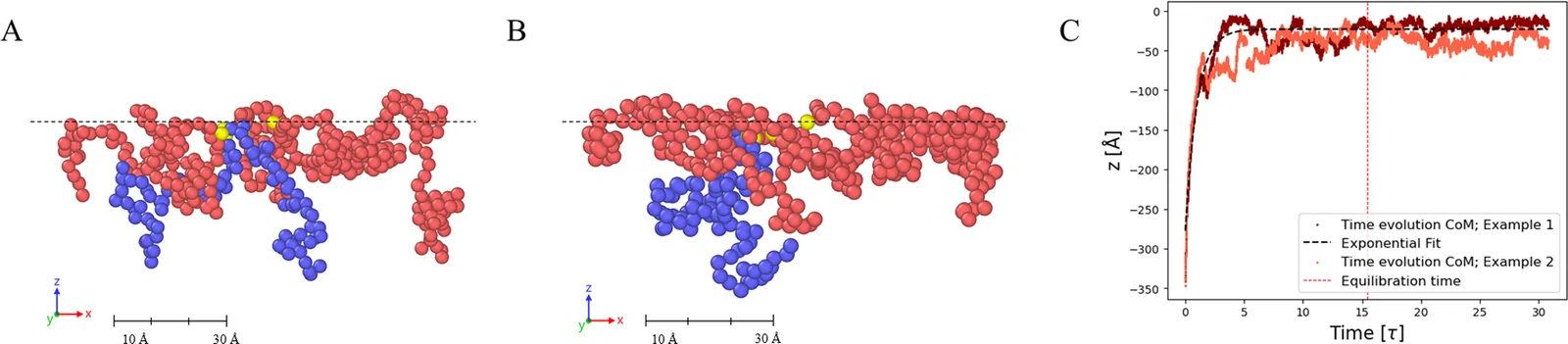
Representative configurations of the cytoplasmic regions of the CD3 complex in simulation (**A**) and (**B**) Two examples of the cytoplasmic regions of the CD3 complex; red units (CD3*ζ* and CD3*ε*) are taken to interact with the plasma membrane in our model; blue units (CD3*γ* and CD3*δ*) do not interact with the plasma membrane; black dotted line indicates the position of the inner leaflet of the plasma membrane. The yellow balls indicate the anchoring points which affix the chains in the membrane. (**C**) Two examples of the time evolution in units of *τ* (characteristic relaxation time constant) of the center of mass in z direction of the *ζ* chain in the five-complex configuration. The vertical dotted line indicates the chosen equilibration time. Data prior to this point were excluded from all subsequent analyses. The black dotted line indicates a fitted saturated exponential function

The polymer chains were initialized in fully extended conformations. To determine an appropriate equilibration time, a simulation was performed using a single complex. An exponential saturation function was fitted to the center-of-mass trajectory, yielding a characteristic relaxation time constant of $\tau = 8.67\times 10^{-7}$ seconds (see fig. 2). The first half of each simulation (the first $5\,\mu \text {s}$, corresponding to ${\approx }\,5.8\tau$) was discarded as equilibration. Only data collected after this equilibration period were used in subsequent analyses. Representative snapshots of the equilibrated single-complex configurations are shown in fig. 2(A) and (B). The scripts for generating input files and running these simulations are provided at (Stevensiegl 2025).

Two configurations were simulated: a single TCR complex (*1-complex*) and five clustered TCR complexes (*5-complex*); see fig. 1. Each configuration was represented as a single simulation box with periodic boundary conditions in *x* and *y* (box dimensions $\pm 736\,$ Å) and a non-periodic boundary in *z* (*-736* to *+50 * Å). The 1-complex system contains 423 beads (6 chains); the 5-complex contains 2115 beads. Dynamics were propagated using Brownian (overdamped Langevin) integration at $T = 323\,\text {K}$ (matching the temperature used in Prakaash et al. (Prakaash et al. 2021)) with a timestep of $1\,\text {fs}$ and a translational friction coefficient $\gamma _t = 1.0$ in LAMMPS real units. Each configuration was run as a single continuous trajectory of $10\,\mu \text {s}$ total; the second half ($5\,\mu \text {s}$) was used for all analyses, which is well past the equilibration period of ${\approx }\,5.8\tau$ ($5\,\mu \text {s}$) established above. Running a single long trajectory rather than multiple short replicas was a deliberate choice to maximise sampling of the slow conformational dynamics of the intrinsically disordered chains.

To calibrate the parameters of the Morse potential, we compared the *z*-positions of the membrane-proximal beads of the *ζ* chain against two reference datasets. Because the Morse potential acts only on beads within its cutoff distance of the membrane (${\sim }100\,$ Å), only the eight most membrane-proximal beads of the *ζ* chain are directly governed by the Morse parameters; the distal tail executes a free random walk whose center-of-mass position reflects chain length rather than membrane affinity. We therefore use the mean *z*-position of these eight proximal beads ($z_\text {prox}$) as our calibration observable. First, we directly analyzed the publicly available coarse-grained trajectories of Prakaash et al. (Prakaash et al. 2021) (5 independent replicas, $5\,\mu \text {s}$ each, with the first $1\,\mu \text {s}$ discarded as equilibration), extracting the $\zeta _1$ cytoplasmic region center of mass relative to the inner leaflet phosphate plane and obtaining $-4.3 \pm 4.4\,$ Å. Our calibrated model (Morse parameters $r_0 = 5.4\,$ Å, $\alpha = 0.050\,$ Å^−1^) reproduces this quantitatively: $z_\text {prox} = -3.5 \pm 5.2\,$ Å. The two distributions agree closely: the means differ by only *0.8 * Å, well within the standard deviation of either, and the effect size is small (Cohen’s *d = 0.16*).

As a second reference, we considered neutron reflection data from Zimmermann et al. (Zimmermann et al. 2017), who report a peak at *15 * Å with a distribution width of approximately *40 * Å; this is an absolute distance from the membrane surface, corresponding to $z \approx -15\,$ Å in our sign convention. Note that $z_\text {prox}$ and the Zimmermann observable are not the same quantity. Our $z_\text {prox}$ captures only the membrane-proximal anchoring of the chain, whereas neutron reflectometry reports the occupied volume of the entire cytoplasmic domain, which naturally extends further from the membrane. The Zimmermann range therefore brackets the full chain extent rather than the proximal anchoring position, and the discrepancy between the two reference values is expected on physical grounds. Quantitatively, *95.9%* of our simulated $z_\text {prox}$ frames fall within the *40 * Å Zimmermann window ($z \in [-35, 5]\,$ Å), confirming consistency. We conducted parameter sweeps and selected parameters that place $z_\text {prox}$ within the Prakaash reference range; the resulting $z_\text {prox}$ distribution and the Prakaash et al. reference are shown in fig. 1(D), with the Zimmermann et al. range indicated by the gray dotted lines. We conclude that our parameter choices are quantitatively consistent with both simulation and experimental data on CD3*ζ* membrane association.

## Results

**Fig. 3:**
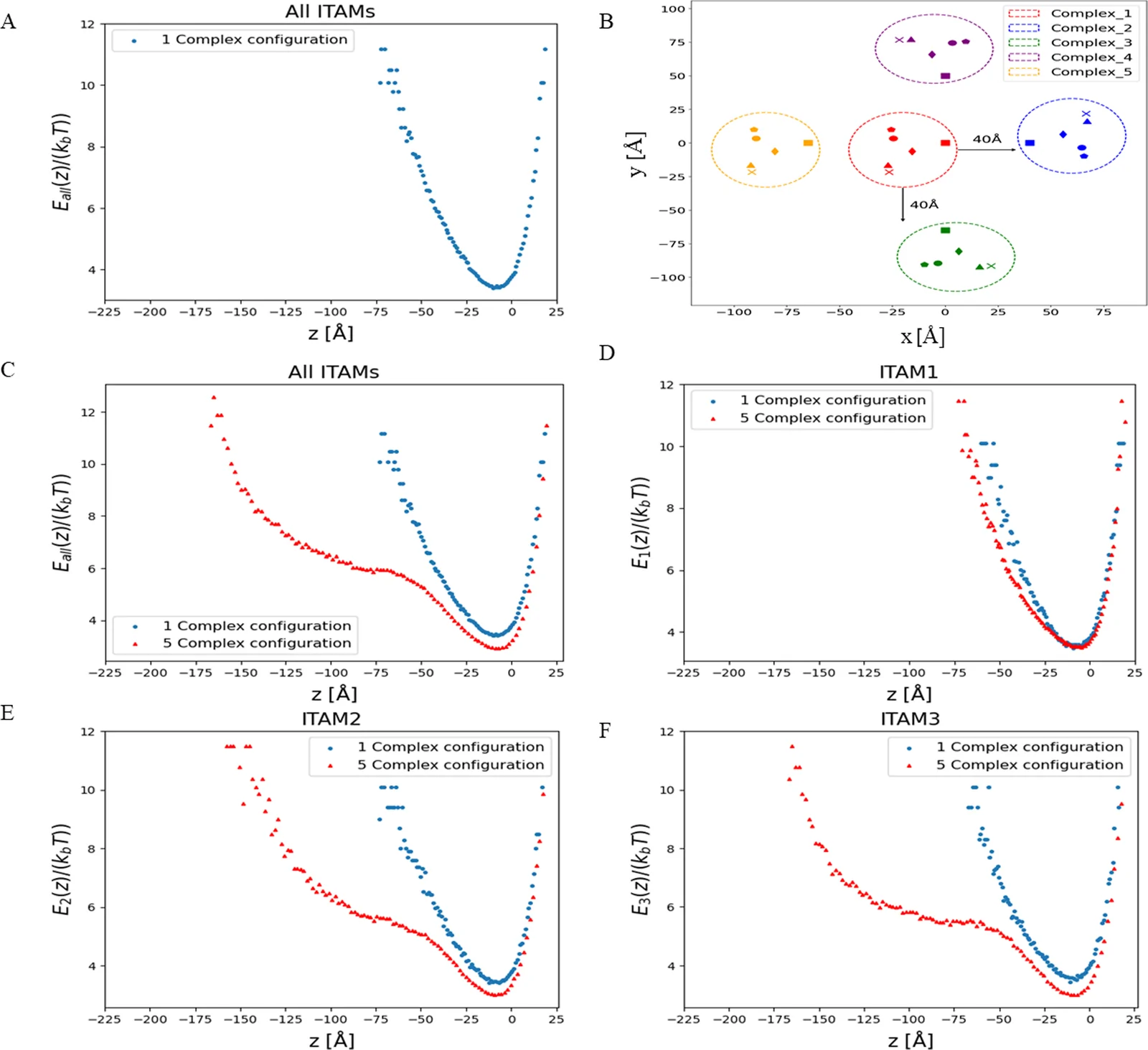
Energy landscape for ITAMs in different configurations (**A**) Energy landscape $E_{all}$ for 1 CD3 complex; (**B**) Fixed relative position of the first unit in the 5 complex configuration; (**C**)-(**F**) Energy landscapes for different ITAMs for the 5 complex configuration (red) and 1 complex configuration (blue); (**C**) Energy landscape $E_{all}$ sum over all ITAMs; (**D**) Energy landscape $E_1$ for the first ITAM; (**E**) Energy landscape $E_2$ for the second ITAM; (**F**) Energy landscape $E_3$ for the third ITAM

To quantify the effect of clustering on CD3*ζ* ITAMs, we introduce the effective energy $E_i(z)$ for the positions of the *i*th ITAM of the CD3$\zeta _\text {cyt}$ along the *z*-axis. It is defined as

1 $$\begin{aligned} E_i = -k_B T \ln p_i , \end{aligned}$$

where $p_i$ is the probability of all microstates associated with the distance *z* of the *i*th ITAM from the membrane, and is taken from simulation. In Fig. 3 we report these energy landscapes for all ITAMs and for the average of all ITAMs, respectively.

The average energy landscape of CD3*ζ* ITAMs, which we define as $E_{all} = \frac{1}{3}\sum _i E_i$, exhibits a single pronounced minimum at *z = 0* (Fig. 3(A)). In the positive *z* direction, the energy rises more steeply compared to the negative *z* direction, preventing the *ζ* chain from reaching the extracellular space.

We next compared the results for ITAMs in CD3$\zeta _\text {cyt}$ with data obtained from a cluster of five TCR complexes (Fig. 3(C)-(F)). The relative positions of the first CD3 units in the cluster configuration are shown in Fig. 3(B). As there is not much information about the TCR arrangement within clusters, we opted here for a regular configuration; the distance between two neighboring protomers within a cluster was assumed to be given by the ectodomain diameter. Between *z = 25 * Å and *z = -25 * Å, the energy profiles for both configurations are nearly identical, with a minimum at *z = 0* (Fig. 3(C)). Beyond *z = -25 * Å, however, the energy landscapes of CD3*ζ* ITAMs in a TCR monomer and in a cluster differ. In the cluster configuration, a kink appears at around *z = -50 * Å, which is absent in the single TCR configuration. Together, the energy profile suggests that clustering does not affect membrane association of ITAMs *per se*. However, it shifts their equilibrium position away from the membrane surface, notably without displacing the most likely position – corresponding to the minimum of the free energy landscape – much. Comparing panels (D)-(F) in Fig. 3, this effect is most pronounced for the energy landscape $E_3$ of the third ITAM, while it is less pronounced for the first-, second- and averaged ITAM energy profile. This likely is a consequence of the fact that the tail end of the chain is the first to be pushed out from the membrane. Notably, for the third ITAM the energy shows a shallow minimum between *z = -45 * Å and *z = -90 * Å, indicating a region of virtually free motion.

**Fig. 4:**
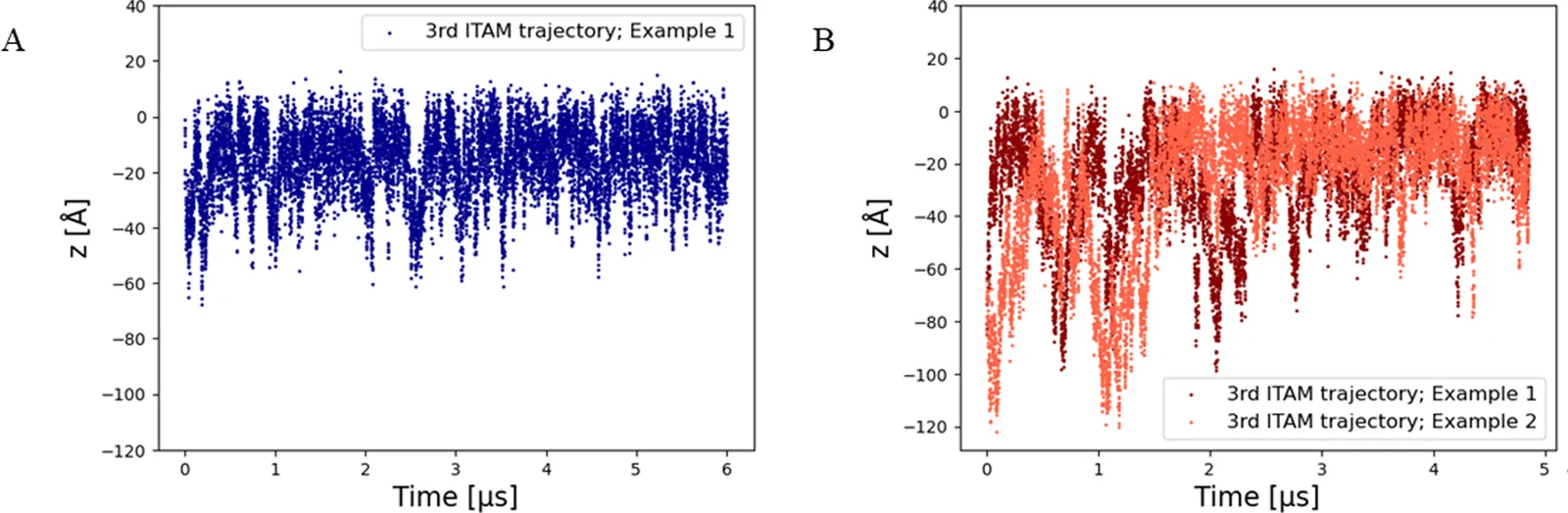
Example trajectories. Representative *z*-direction trajectory for all 3 ITAMs (**A**) in the one-complex configuration; (**B**) and in the five-complex configuration, shown after the equilibration period. Time zero corresponds to the start of data collection following system equilibration

**Fig. 5:**
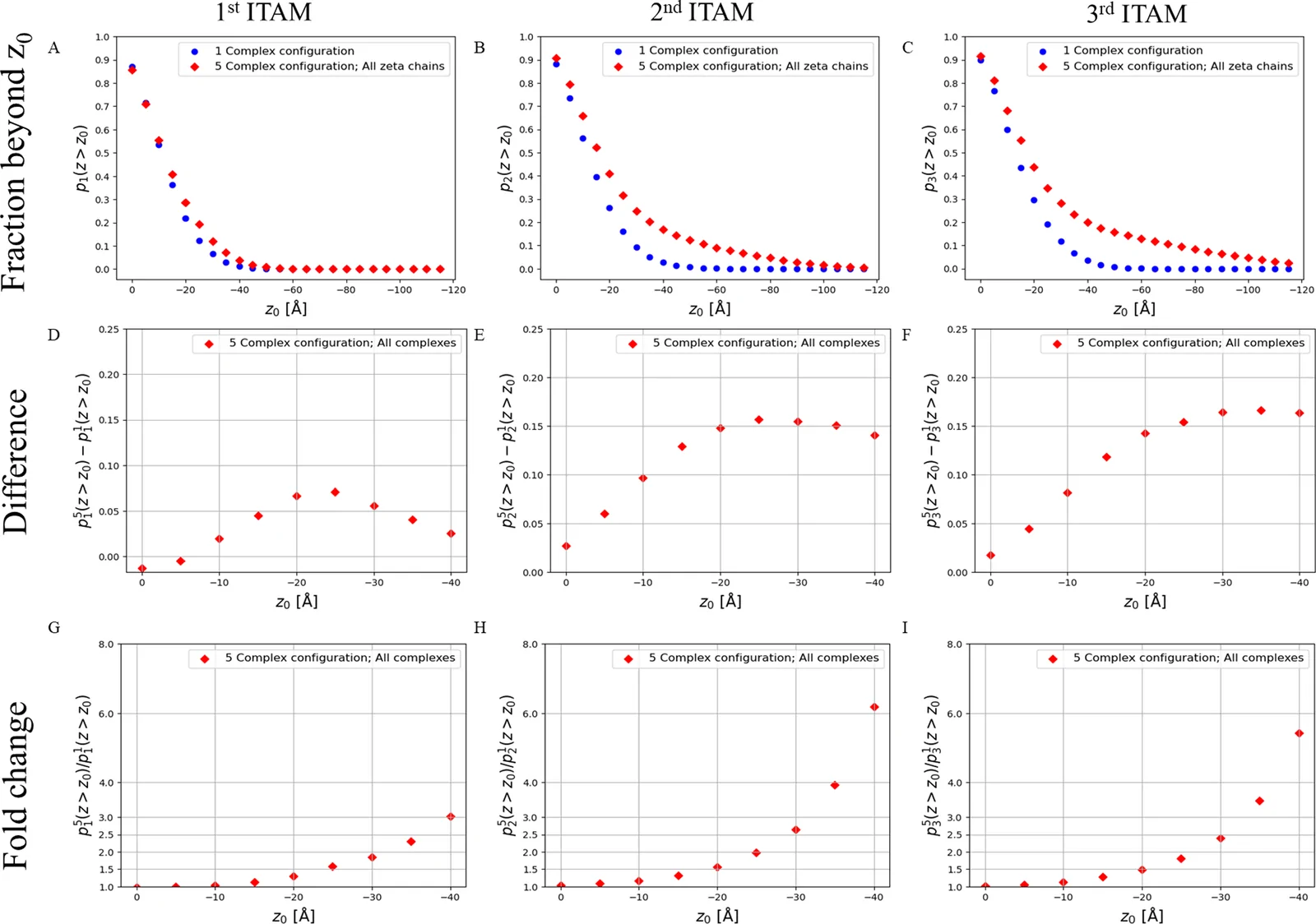
Fraction of cumulative time ITAMs spend beyond a certain threshold distance (**A**)-(**C**) $p_i(z>z_0)$ versus $z_0$ for the ith CD3*ζ* ITAM. This plot compares the single TCR configuration (blue) with the five-TCR configuration (red) for the (**A**) 1 st; (**B**) 2nd; (**C**) 3rd ITAM; (**D**)-(**F**) Difference of $p_i(z>z_0)$ between the single TCR configuration and the five-TCR configuration for the (**D**) 1 st; (**E**) 2nd; (**F**) 3rd ITAM; (**G**)-(**I**) Fold change of $p_i(z>z_0)$ between the five-TCR configuration and the single TCR configuration for the (**G**) 1 st; (**H**) 2nd; (**I**) 3rd ITAM. Panels (**G**)-(**I**) are shown on a logarithmic scale to accommodate the large dynamic range of the fold change across threshold distances

While the free energy landscape shows that ITAMs tend to be more distant from the membrane in case the TCR complex is clustered, it does not provide information on ITAM membrane dissociation-association dynamics. To better understand these dynamics, in Fig. 4 we show representative trajectories of the third CD3*ζ* ITAM. Together with the energy landscapes, visual inspection of such trajectories led us to hypothesize that the duration of ITAM excursions to distal regions from the membrane would be notably increased in the 5-complex configuration. To quantify this effect, we determined the fraction of total simulation time during which the three different CD3*ζ* ITAMs were located at distances exceeding a certain separation from the membrane, $z_0$, $p_i(z>z_0)$ (fFig. 5(A)-(C)). Irrespective of the TCR configuration, ITAM-1 is found near the membrane ($z_0 = 10 \,$ Å) for about *30%* of the time, ITAM-2 and ITAM-3 for about *20%* of the time (Fig. 5(B)-(C)). This is consistent with our earlier finding that the free energy landscape minima of ITAM-2 and ITAM-3 did not shift between the 1-complex and 5-complex configurations (cf. Fig. 3(E)-(F)).

The differences in probabilities between the 5-complex and 1-complex configuration show pronounced maxima for each ITAM. The positions of the maxima range from around $z_0 = -25 \,$ Å for the first ITAM to around $z_0 = -35 \,$ Å for the third ITAM Fig. 5(D)-(F). In words, the likelihood of finding ITAM-3 at this distance is 16.5 % higher in the 5-complex configuration than in the 1-complex configuration. The difference between the two configurations, however, becomes much more apparent when plotting the ratio of the two probabilities Fig. 5(G)-(I): Across the range $z_0 = -20$ – *-40 * Å, the five-complex configuration consistently shows a more than 1.5-fold higher fraction of time spent beyond each threshold distance compared to the single-complex configuration.

### Robustness of results with respect to cluster geometry

To assess whether the qualitative findings depend on the precise lateral arrangement of protomers within the cluster, we performed an additional simulation with a *rotationally symmetric* configuration in which all five complexes are placed as pure rotational copies of a single reference complex. The ITAM energy landscapes and cumulative probability curves from this configuration are provided as Supplementary Figures S1 and S2 (see the separate Supplementary Information document); all qualitative findings are reproduced: clustering induces a kink in the energy landscape at $z \approx -50\,$ Å, and the cumulative probability ratio exceeds 1.5-fold across the range $z_0 = -20$ to *-40 * Å. We conclude that the stretching effect is an intrinsic consequence of steric crowding between the cytoplasmic chains and is not sensitive to the precise lateral arrangement of protomers within the cluster.

## Discussion

Numerous experimental observations have shown that TCR crosslinking, by antibodies or artificially in-vitro, can induce intracellular T-cell signaling. Once activated, the cascade of events that follow is largely indistinguishable from antigen-specific activation (Menon et al. 2023; Spencer et al. 1993; Ma et al. 2020; Letourneur and Klausner 1991). Importantly, neither monovalent ligands nor soluble pMHC reveal T-cell activation. Among antibodies, those against CD3*ε* are most potent. Since CD3*ε* is the only subunit with more than one copies per TCR complex, these data are commonly interpreted as indication that TCR clustering – and not only dimerization – is important for activation. Interestingly, we have previously found that during pMHC-mediated antigen recognition TCRs do not show detectable oligomerization (Brameshuber et al. 2018). Together, these findings suggest that the two signal transduction processes – T-cell activation via TCR crosslinking and via antigen-specific activation – share common consequences in signaling, albeit employing different transduction mechanisms. Indeed, we previously reported that by artificially preventing TCR clustering during triggering via a single-chain antibody fragment – using DNA nanostructures – we could efficiently abrogate T-cell signaling; conversely, antigen-specific triggering via pMHC on the same DNA nanostructures had no effect on signaling (Hellmeier et al. 2021). In summary, TCR clustering appears to be at least a critical transient intermediate during TCR-mediated T-cell activation.

In this manuscript, we addressed the question whether TCR clustering *per se* would suffice to induce a conformational change that affects downstream signaling, using coarse-grained molecular dynamics simulations of the TCR complex alone or in a pentameric arrangement. Based on previous literature showing that CD3*ζ* clustering triggers T-cell activation, and given the fact that CD3*ζ* has by far the largest cytoplasmic domain, we opted here for an analysis of clustering-induced conformational changes of CD3*ζ*. In solution, the cytoplasmic domains of CD3*ζ* are non-structured and can therefore be classified as intrinsically disordered proteins. However, unlike standard intrinsically disordered soluble proteins, CD3$\zeta _\text {cyt}$ is not only linked through the CD3*ζ* transmembrane region to the plasma membrane, it also changes its local concentration during the T-cell activation process as a consequence of the clustering of TCR. These features are reminiscent of grafting polymers to surfaces to form polymer brushes. Depending on the polymer surface density in relation to its radius of gyration, different regimes in brush formation can be discriminated (Brittain and Minko 2007): In the weakly interacting regime (also called mushroom regime), chains are grafted at distances larger than the radius of gyration, and polymers can be treated as isolated surface-anchored molecules. In this regime, the layer thickness is independent of the grafting density. In the polymer brush regime, the layer thickness increases with grafting density, finally transiting to the highly stretched regime, where the polymer brush reaches thicknesses of up to 70% of the polymer’s contour length. While polymers in the weakly interacting regime typically form isolated three-dimensional mushrooms, the highly amphiphilic character of CD3$\zeta _\text {cyt}$ leads to its confinement to the two-dimensional surface of the cytosolic leaf of the plasma membrane, further increasing local crowding of CD3$\zeta _\text {cyt}$ upon TCR clustering. Taking additionally into account the presence of two membrane-associated CD3*ε* domains per TCR, we can describe the observed stretching of CD3$\zeta _\text {cyt}$ as a clustering-induced transition from the weakly interacting regime to the polymer brush regime, thereby shifting the positions of its ITAMs deeper into the cytoplasm.

**Fig. 6:**
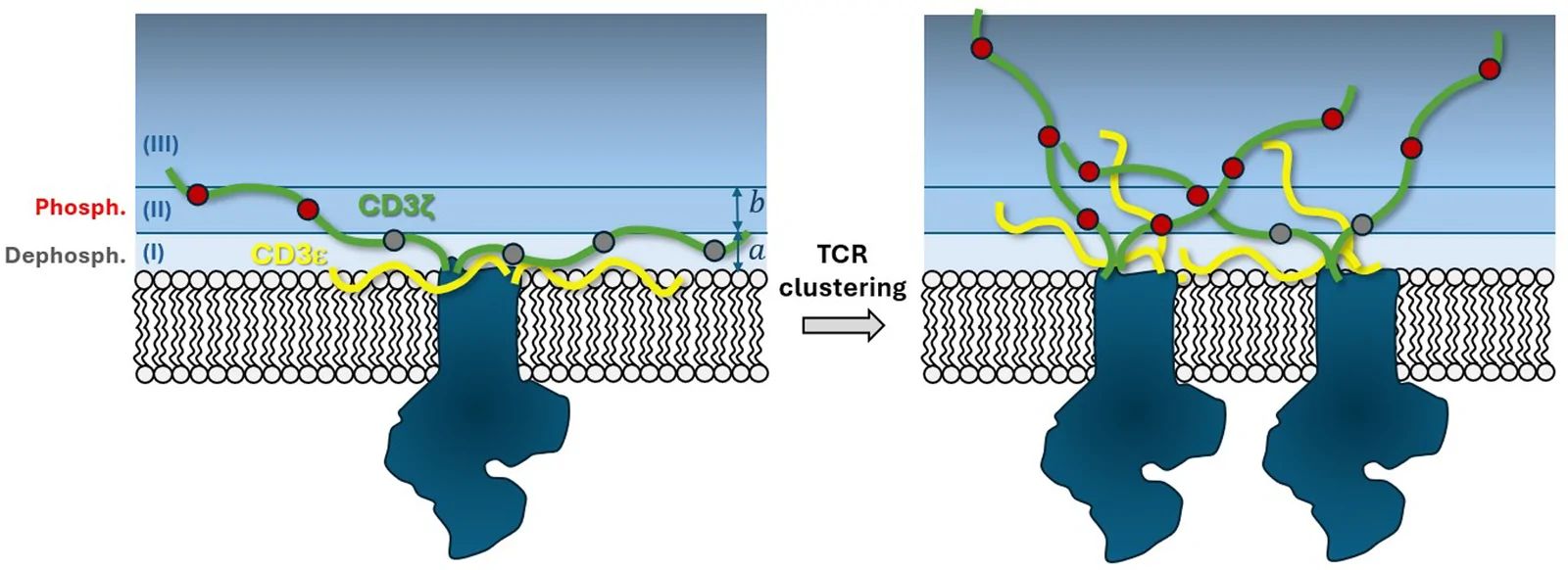
Model of clustering-induced CD3*ζ*ITAM phosphorylation. The TCR is sketched according to Dong et al. (2019). For clarity, we only included the cytoplasmic CD3*ζ* (green) and CD3*ε* chains (yellow). Phosphorylated and dephosphorylated ITAMs are shown as red or grey circles, respectively. Layers (I), (II) and (III) are indicated in different shades of blue. (left) Without TCR clustering, CD3*ζ* ITAMs are mainly located in layer (I), where CD45-mediated ITAM dephosphorylation dominates. Residual phosphorylation via lck or fyn occurs when ITAMs translocate to layer (II). (right). TCR clustering leads to a shift in the average ITAM positions towards layer (II), where phosphorylation dominates

In general, we observed random dissociation of CD3*ζ* ITAMs from and association events with the membrane, both in the clustered and non-clustered configuration. In the clustered configuration, however, these dissociation events shift the ITAMs on average further away from the membrane than in the monomeric configuration. The consequences of this clustering-induced stretch for T-cell activation might be profound. To demonstrate this, we posit a simple model with 3 characteristic layers (fig. 6): if ITAMs reside in a membrane-proximal layer (I) of width *a*, they are preferentially dephosphorylated by the phosphatase CD45. In contrast, phosphorylation by lck or fyn dominates in the adjacent layer (II) (width *b*). Finally, the most distal layer (III) is out of reach for both phosphorylation and dephosphorylation processes, leaving the ITAM in the same state it featured when transiting to layer (III). The values of *a* and *b* are set by the geometry of the relevant enzymes: the membrane-proximal catalytic domain of CD45 reaches approximately *20 * Å from the inner leaflet, giving $a \approx 20\,$ Å; the kinases lck and fyn, when fully extended from their membrane anchors, place their catalytic domains at approximately *30 * Å, giving $b \approx 30\,$ Å (see below for details and references). The sensitivity of the predicted fold-change to variations in *a* and *b* can be read directly from fig. 5(G)–(I): shifting either cutoff by $\pm 5\,$ Å changes the absolute fold-change values but does not alter the qualitative conclusion that clustering enhances ITAM phosphorylation by a factor of 1.5–2. Therefore, if the according rates for (de-)phosphorylation are sufficiently fast, all ITAMS residing in layer (I) will be dephosphorylated, whereas all ITAMs in layers (II) and (III) will be phosphorylated. In this simple model, the average degrees of ITAM-i phosphorylation is given by the probability distribution $p_i(z>a)$, and the consequences of CD3*ζ* clustering by the ratio $\frac{p^{(clustered)}_i(z>a)}{p^{(monomer)}_i(z>a)}$, where superscript denotes the clustered or monomeric TCR. This ratio has been plotted in Fig. 5(G)-(I).

Let us relate these general findings to data known for the key signaling proteins CD45, lck and fyn. The phosphatase CD45 has two cytoplasmic domains, one of which – the most membrane-proximal – shows phosphatase activity, whereas the second domain is inactive (Poy et al. 2005). The membrane-distance of the catalytic site is appr. *20  * Å, setting in our model $a \approx 20 \,$ Å. The kinase lck is a 56 kD protein, which is associated with the inner leaflet of the T-cell plasma membrane by various means: i) it binds to the co-receptor CD4 or CD8; ii) it has three acylation motifs; iii) its SH2 domain interacts with inositol-lipids (Prakaash et al. 2022). It was found that lck may adopt different configurations, ranging from a membrane-associated to a distal configuration when fully stretched; the latter corresponds to a distance between membrane surface and catalytic domain of appr. *30 * Å. The kinase fyn is a 59 kD protein, with three acylation sites facilitating its membrane association (Gerbec et al. 2015). Similarities in the domain structure of lck and fyn impose similar constraints on the distance of ITAMs from the membrane surface. In our three layer model, hence, we can set $b \approx 30 \,$ Å. From fig. 5(G)-(I) we can thus conclude that, in our simple model, we expect a 2.5-fold increased phosphorylation degree of ITAM-1 and ITAM-2, and a 2-fold increased phosphorylation degree of ITAM-3 upon TCR clustering.

Our coarse-grained model uses a uniform Lennard-Jones excluded-volume potential and a residue-agnostic Morse membrane interaction. More sophisticated force fields for intrinsically disordered proteins - such as CALVADOS (Tesei et al. 2021), which employs sequence-specific Ashbaugh–Hatch hydrophobicity parameters and explicit Debye–Hückel electrostatics - could in principle capture sequence-level effects on chain compaction and membrane affinity that our model neglects. The advantage of our simpler approach is that it isolates the purely steric crowding effect of clustering from sequence-specific contributions, making the physical mechanism transparent: the stretching we observe arises solely from excluded-volume repulsion between chains, not from electrostatic or hydrophobic details. A natural extension of this work would be to test whether the qualitative conclusions hold in a CALVADOS-type model that includes electrostatics and sequence specificity.

One obvious limitation of our study is that it employs a coarse-grained model, which neglects many effects that are likely to influence interaction ranges and repulsion between the intracellular chains of the TCR. In particular, CD3*ζ* chains are partly positively charged due to the presence of basic-rich regions. The static interactions modeled here are rather short-ranged compared to electrostatic (Coulombic) interactions. Thus, our work is likely to underestimate the effects of clustering on chain stretching. Furthermore, while our simulations used clusters of five CD3 complexes, it would be valuable to explore larger clusters that are more representative of those observed in experimental systems (Yokosuka et al. 2005; Varma et al. 2006; Campi et al. 2005). It is plausible that larger clusters could further amplify the observed effects.

Together, the simulations presented here serve as an indication that clustering of CD3 complexes could facilitate TCR activation through steric interactions between intracellular domains.

## Electronic Supplementary Material

Below is the link to the electronic supplementary material.

Supplementary Material 1

## Data Availability

The code used to generate the results in this study is available for download at https://github.com/StevenSiegl/CD3_lammps.

## References

[CR1] Aivazian D, Stern LJ (2000) Phosphorylation of t cell receptor is regulated by a lipid dependent folding transition. Nat Struct Biol 7(11):1023–1026 10.1038/80930 11062556

[CR2] Brameshuber M, Kellner F, Rossboth BK, Ta H, Alge K, Sevcsik E, Göhring J, Axmann M, Baumgart F, Gascoigne NR et al (2018) Monomeric tcrs drive t cell antigen recognition. Nat Immunol 19(5):487–496 10.1038/s41590-018-0092-4 PMC7612939 29662172

[CR3] Brittain WJ, Minko S (2007) A structural definition of polymer brushes. J Polym Sci, Part A: Polym Chem 45(16):3505–3512

[CR4] Call ME, Schnell JR, Xu C, Lutz RA, Chou JJ, Wucherpfennig KW (2006) The structure of the transmembrane dimer reveals features essential for its assembly with the t cell receptor. Cell 127(2):355–368 10.1016/j.cell.2006.08.044 PMC3466601 17055436

[CR5] Campi G, Varma R, Dustin ML (2005) Actin and agonist MHC-peptide complex-dependent t cell receptor microclusters as scaffolds for signaling. J Exp Med 202(8):1031–1036 10.1084/jem.20051182 PMC1373686 16216891

[CR6] Davis SJ, Van Der Merwe PA (2006) The kinetic-segregation model: Tcr triggering and beyond. Nat Immunol 7(8):803–809 10.1038/ni1369 16855606

[CR7] Dong D, Zheng L, Lin J, Zhang B, Zhu Y, Li N, Xie S, Wang Y, Gao N, Huang Z (2019) Structural basis of assembly of the human t cell receptor-cd3 complex. Nature 573(7775):546–552 10.1038/s41586-019-1537-0 31461748

[CR8] Gascoigne NR, Casas J, Brzostek J, Rybakin V (2011) Initiation of tcr phosphorylation and signal transduction. Front Immunol 2:72 10.3389/fimmu.2011.00072 PMC3342367 22566861

[CR9] Gerbec ZJ, Thakar MS, Malarkannan S (2015) The fyn-adap axis: cytotoxicity versus cytokine production in killer cells. Front Immunol 6:472 10.3389/fimmu.2015.00472 PMC4584950 26441977

[CR10] Grakoui A, Bromley SK, Sumen C, Davis MM, Shaw AS, Allen PM, Dustin ML (1999) The immunological synapse: a molecular machine controlling t cell activation. Science 285(5425):221–227 10.1126/science.285.5425.221 10398592

[CR11] Hellmeier J, Platzer R, Eklund A, Schlichthaerle T, Schuetz GJ, Jungmann R, Huppa JB, Sevcsik E (2021) Dna origami demonstrate the unique stimulatory power of single pmhcs as t-cell antigens. Biophys J 120(3):330 10.1073/pnas.2016857118 PMC7848602 33468643

[CR12] Letourneur F, Klausner RD (1991) T-cell and basophil activation through the cytoplasmic tail of t-cell-receptor zeta family proteins. Proc Natl Acad Sci U S A 88(20):8905–8909 10.1073/pnas.88.20.8905 PMC52619 1833767

[CR13] Love PE, Hayes SM (2010) Itam-mediated signaling by the t-cell antigen receptor. Cold Spring Harb Perspect Biol 2(6):002485 10.1101/cshperspect.a002485 PMC2869518 20516133

[CR14] Ma Y, Lim YJ, Benda A, Lou J, Goyette J, Gaus K (2020) Clustering of the -chain can initiate t cell receptor signaling. Int J Mol Sci 21(10):3498 10.3390/ijms21103498 PMC7279048 32429097

[CR15] Menon A, Moreno B, Meraviglia-Crivelli D, Nonatelli F, Villanueva H, Barainka M, Zheleva A, Santen H, Pastor F (2023) Modulating T Cell Responses by Targeting CD3. Cancers 15:1189 10.3390/cancers15041189 PMC9953819 36831533

[CR16] Van Der Merwe PA, Dushek O (2011) Mechanisms for t cell receptor triggering. Nat Rev Immunol 11(1):47–55 10.1038/nri2887 21127503

[CR17] Poy NH-J, Saito F, Frederick H, CA, (2005) Structural basis for the function and regulation of the receptor protein tyrosine phosphatase cd45. J Exp Med 201(3):441–452 10.1084/jem.20041890 PMC2213029 15684325

[CR18] Prakaash D, Cook GP, Acuto O, Kalli AC (2021) Multi-scale simulations of the t cell receptor reveal its lipid interactions, dynamics and the arrangement of its cytoplasmic region. PLoS Comput Biol 17(7):1009232 10.1371/journal.pcbi.1009232 PMC8321403 34280187

[CR19] Prakaash D, Fagnen C, Cook GP, Acuto O, Kalli AC (2022) Molecular dynamics simulations reveal membrane lipid interactions of the full-length lymphocyte specific kinase (LCK). Sci Rep 12(1):21121 10.1038/s41598-022-25603-6 PMC9729596 36476673

[CR20] Saotome K, Dudgeon D, Colotti K, Moore MJ, Jones J, Zhou Y, Rafique A, Yancopoulos GD, Murphy AJ, Lin JC et al (2023) Structural analysis of cancer-relevant tcr-cd3 and peptide-mhc complexes by cryoem. Nat Commun 14(1):2401 10.1038/s41467-023-37532-7 PMC10132440 37100770

[CR21] Sasmal DK, Feng W, Roy S, Leung P, He Y, Cai C, Cao G, Lian H, Qin J, Hui E et al (2020) Tcr-pmhc bond conformation controls tcr ligand discrimination. Cell Mol Immunol 17(3):203–217 10.1038/s41423-019-0273-6 PMC7052167 31530899

[CR22] Sigalov AB, Aivazian DA, Uversky VN, Stern LJ (2006) Lipid-binding activity of intrinsically unstructured cytoplasmic domains of multichain immune recognition receptor signaling subunits. Biochemistry 45(51):15731–15739 10.1021/bi061108f PMC2528957 17176095

[CR23] Spencer DM, Wandless TJ, Schreiber SL, Crabtree GR (1993) Controlling signal transduction with synthetic ligands. Science 262(5136):1019–1024 10.1126/science.7694365 7694365

[CR24] Sušac L, Vuong MT, Thomas C, Bülow S, O’Brien-Ball C, Santos AM, Fernandes RA, Hummer G, Tampé R, Davis SJ (2022) Structure of a fully assembled tumor-specific t cell receptor ligated by pmhc. Cell 185(17):3201–3213 10.1016/j.cell.2022.07.010 PMC9630439 35985289

[CR25] StevenSiegl: CD3_lammps (2025). https://github.com/StevenSiegl/CD3_lammps

[CR26] Tesei G, Schulze TK, Crehuet R, Lindorff-Larsen K (2021) Accurate model of liquid-liquid phase behavior of intrinsically disordered proteins from optimization of single-chain properties. Proc Natl Acad Sci U S A 118(44):2111696118 10.1073/pnas.2111696118 PMC8612223 34716273

[CR27] Thompson AP, Aktulga HM, Berger R, Bolintineanu DS, Brown WM, Crozier PS, In’t Veld PJ, Kohlmeyer A, Moore SG, Nguyen TD et al (2022) Lammps-a flexible simulation tool for particle-based materials modeling at the atomic, meso, and continuum scales. Comput Phys Commun 271:108171

[CR28] Varma R, Campi G, Yokosuka T, Saito T, Dustin ML (2006) T cell receptor-proximal signals are sustained in peripheral microclusters and terminated in the central supramolecular activation cluster. Immunity 25(1):117–127 10.1016/j.immuni.2006.04.010 PMC1626533 16860761

[CR29] Wang H, Kadlecek TA, Au-Yeung BB, Goodfellow HES, Hsu LY, Freedman TS, Weiss A (2010) Zap-70: an essential kinase in t-cell signaling. Cold Spring Harb Perspect Biol 2(5):a002279 10.1101/cshperspect.a002279 PMC2857167 20452964

[CR30] Wucherpfennig KW, Gagnon E, Call MJ, Huseby ES, Call ME (2010) Structural biology of the t-cell receptor: insights into receptor assembly, ligand recognition, and initiation of signaling. Cold Spring Harb Perspect Biol 2(4):005140 10.1101/cshperspect.a005140 PMC2845206 20452950

[CR31] Xu C, Gagnon E, Call ME, Schnell JR, Schwieters CD, Carman CV, Chou JJ, Wucherpfennig KW (2008) Regulation of t cell receptor activation by dynamic membrane binding of the cd3 cytoplasmic tyrosine-based motif. Cell 135(4):702–713 10.1016/j.cell.2008.09.044 PMC2597348 19013279

[CR32] Yokosuka T, Sakata-Sogawa K, Kobayashi W, Hiroshima M, Hashimoto-Tane A, Tokunaga M, Dustin ML, Saito T (2005) Newly generated t cell receptor microclusters initiate and sustain t cell activation by recruitment of zap70 and slp-76. Nat Immunol 6(12):1253–1262 10.1038/ni1272 16273097

[CR33] Zhang H, Cordoba SP, Dushek O, Anton Van Der Merwe P (2011) Basic residues in the t-cell receptor cytoplasmic domain mediate membrane association and modulate signaling. *Proceedings of the National Academy of Sciences* **108**(48), 19323–19328 10.1073/pnas.1108052108 PMC3228420 22084078

[CR34] Zhao B, Brittain WJ (2000) Polymer brushes: surface-immobilized macromolecules. Prog Polym Sci 25(5):677–710

[CR35] Zimmermann K, Eells R, Heinrich F, Rintoul S, Josey B, Shekhar P, Lösche M, Stern LJ (2017) The cytosolic domain of t-cell receptor associates with membranes in a dynamic equilibrium and deeply penetrates the bilayer. J Biol Chem 292(43):17746–17759 10.1074/jbc.M117.794370 PMC5663876 28893902

