# Supplementary material for: Clustering of the T-cell receptor complex may facilitate T-cell activation by stretching of CD3ζ cytoplasmic tails
Source: Eur Biophys J. 2026 Jul 27;55(5):887–96. doi: 10.1007/s00249-026-01861-4 (PMC13661662; doi:10.1007/s00249-026-01861-4)
Supplement: Supplementary file 1 [file 249_2026_1861_MOESM1_ESM.pdf]

Supplementary Information for: Clustering of the T-cell receptor complex may facilitate T-cell activation by stretching of CD3ζ cytoplasmic tails

Steven Siegl[1], Sebastian Fürthauer[1*], Gerhard J. Schütz[1*]

[1*]Institute of Applied Physics, TU Wien, Lehargasse 6, Vienna, 1060, Austria.

*Corresponding author(s). E-mail(s): fuerthauer@iap.tuwien.ac.at; schuetz@iap.tuwien.ac.at;

Robustness with respect to cluster geometry

In the main text, the five complexes of the cluster were arranged on a regular grid, with the inter-protomer spacing set by the ectodomain diameter. Because little is known about the precise lateral organisation of TCRs within a cluster, we tested whether the stretching effect depends on this particular arrangement. To this end, we repeated the five-complex simulation using a rotationally symmetric configuration, in which the five complexes are generated as pure rotational copies of a single reference complex about the cluster centre.

The resulting ITAM energy landscapes are shown in fig. S1, and the corresponding cumulative-probability analysis in fig. S2. Both reproduce the features observed for the grid-like arrangement: the energy landscapes develop a kink at $z \approx -50$ Å that is absent in the single-complex reference, and the cumulative-probability ratio between the clustered and monomeric configurations exceeds 1.5 across the range $z_0 = -20$ to -40 Å. The close quantitative agreement between the two geometries indicates that the clustering-induced stretch is governed by steric crowding among the cytoplasmic chains and is essentially insensitive to the specific lateral packing of protomers within the cluster.

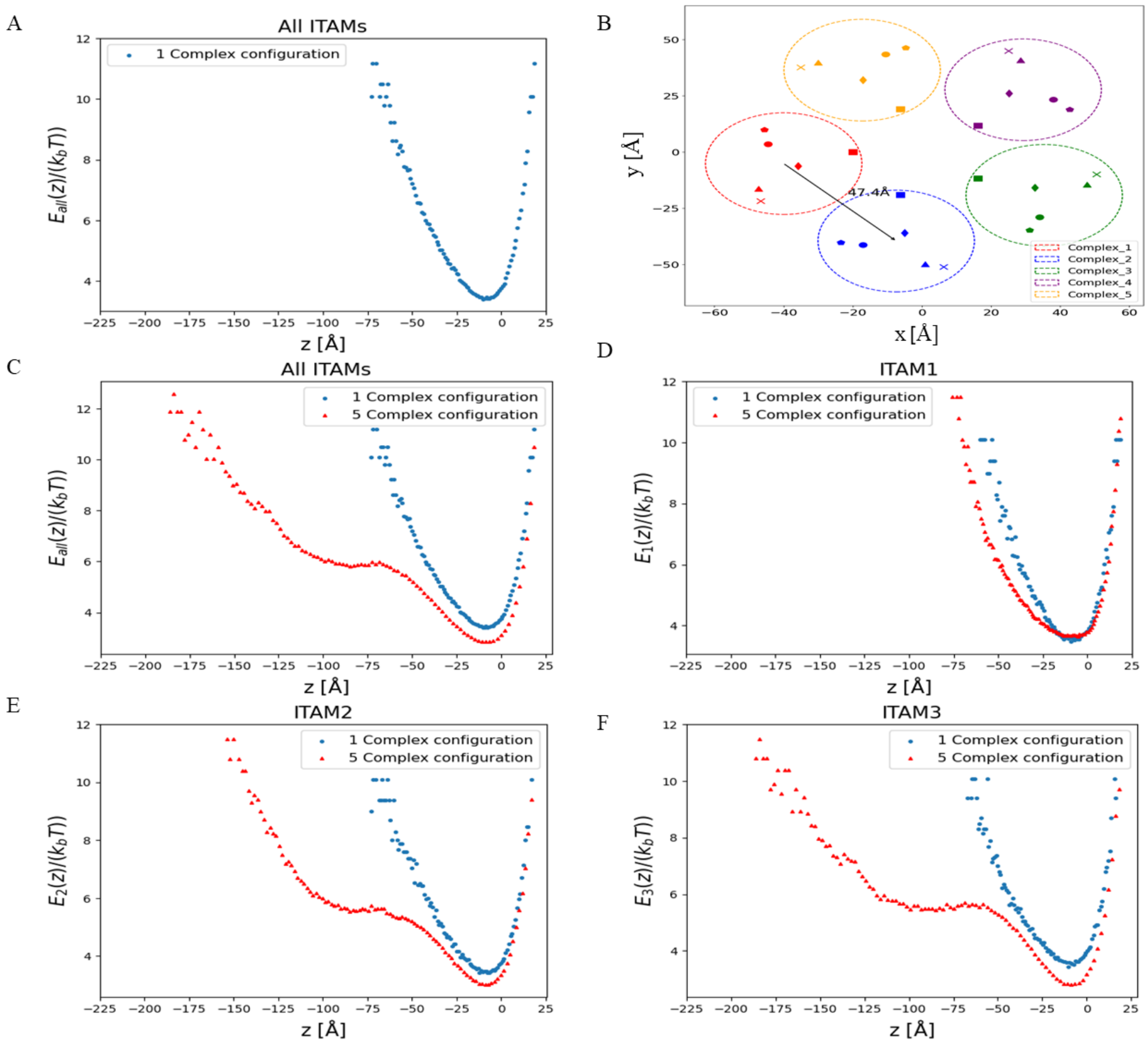

Fig. S1 Energy landscape for ITAMs in different configurations (A) Energy landscape E_{all} for 1 CD3 complex; (B) Fixed relative position of the first unit in the 5 complex configuration;(C)-(F) Energy landscapes for different ITAMs for the 5 complex configuration (red) and 1 complex configuration (blue); (C) Energy landscape E_{all} sum over all ITAMs; (D) Energy landscape E_1 for the first ITAM; (E) Energy landscape E_2 for the second ITAM; (F) Energy landscape E_3 for the third ITAM.

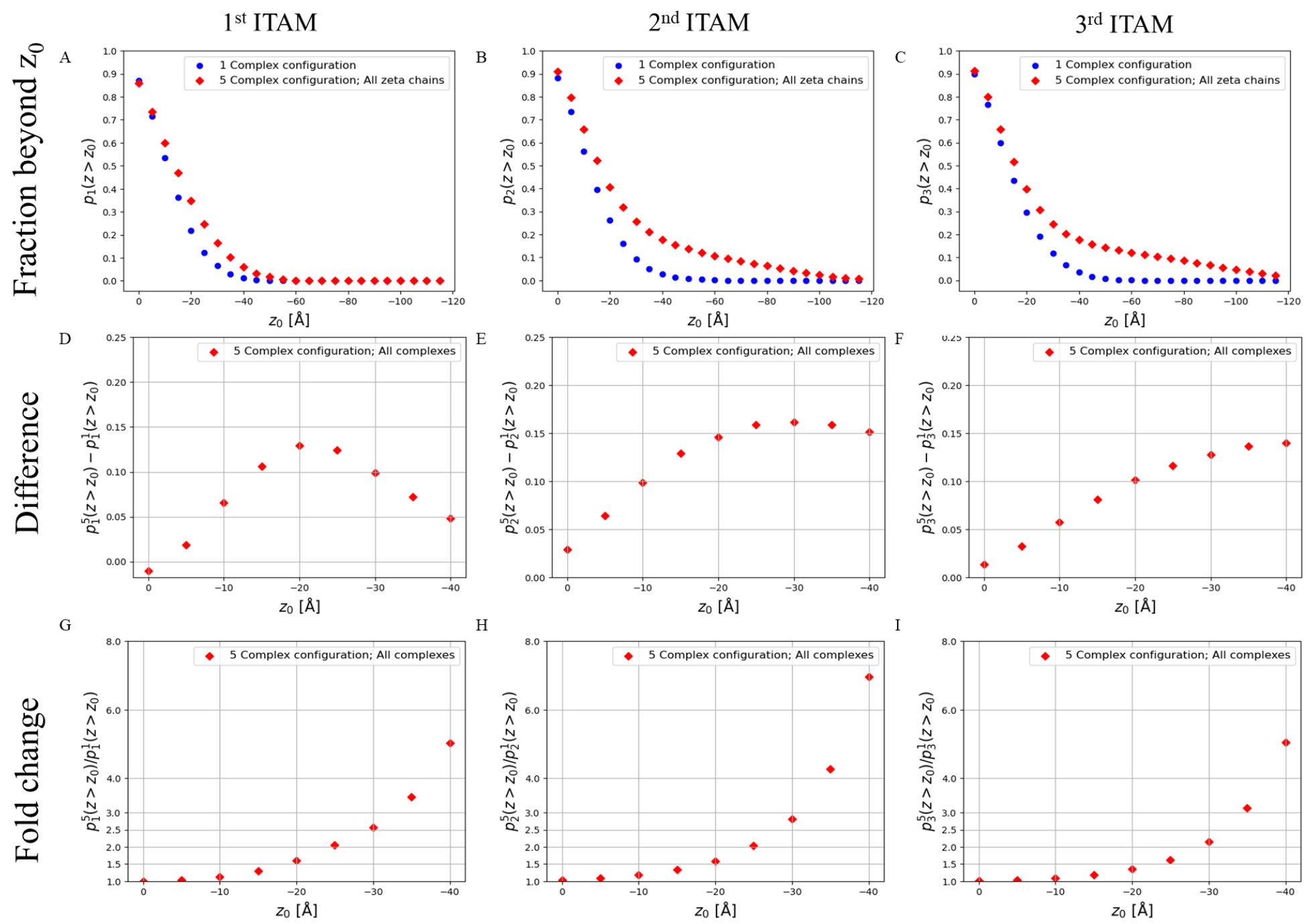

Fig. S2 Fraction of cumulative time ITAMs spend beyond a certain threshold distance (A)-(C) $p_i(z > z_0)$ versus z_0 for the ith CD3ζ ITAM. This plot compares the single TCR configuration (blue) with the five-TCR configuration (red) for the (A) 1st; (B) 2nd; (C) 3rd ITAM; (D)-(F) Difference of $p_i(z > z_0)$ between the single TCR configuration and the five-TCR configuration for the (D) 1st; (E) 2nd; (F) 3rd ITAM; (G)-(I) Fold change of $p_i(z > z_0)$ between the five-TCR configuration and the single TCR configuration for the (G) 1st; (H) 2nd; (I) 3rd ITAM. Panels (G)-(I) are shown on a logarithmic scale to accommodate the large dynamic range of the fold change across threshold distances.

Statistical convergence: block averaging

Because each configuration was sampled with a single long trajectory rather than with independent replicas, we estimated the statistical uncertainty of the energy landscapes by block averaging. The production part of each trajectory was divided into ten consecutive, non-overlapping blocks of equal length, and the energy landscape $E(z)/k_{\mathrm{B}}T$ was computed independently for each block (thin light-blue lines). The dark-blue curve denotes the mean across blocks and the shaded band indicates ± 1 standard deviation.

Figures S3 to S5 show this analysis for the single-complex (1C), grid-like five-complex (5C), and rotationally symmetric five-complex configurations, respectively. In all cases the block-to-block scatter is smallest near the free-energy minimum ($z \approx 0$), where the chains are sampled most densely, and grows in the distal, high-energy tails, which are visited only rarely and are therefore less well converged. Crucially, the conclusions drawn in the main text rest on the well-sampled region around the minimum and on the onset of the kink, where the standard deviation remains small

compared with the differences between configurations. The block-averaging analysis thus confirms that the reported features are statistically robust and not artefacts of limited sampling.

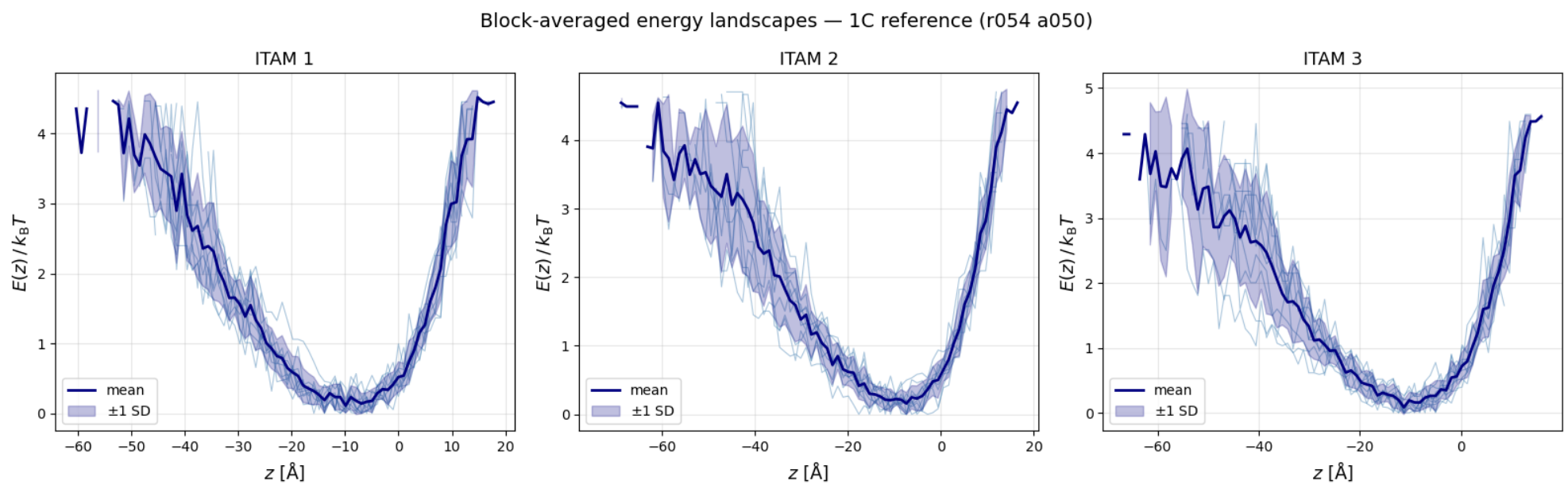

Fig. S3 Block-averaged energy landscapes for the single-complex (1C) configuration. Potential of mean force, $E(z)/k_{\mathrm{B}}T$, as a function of the reaction coordinate z for the three ITAM motifs (ITAM 1–3). Simulation data were divided into 10 blocks, and the energy landscape of each block is shown as a thin light-blue line. The dark-blue curve denotes the mean across blocks, and the shaded band indicates ±1 standard deviation

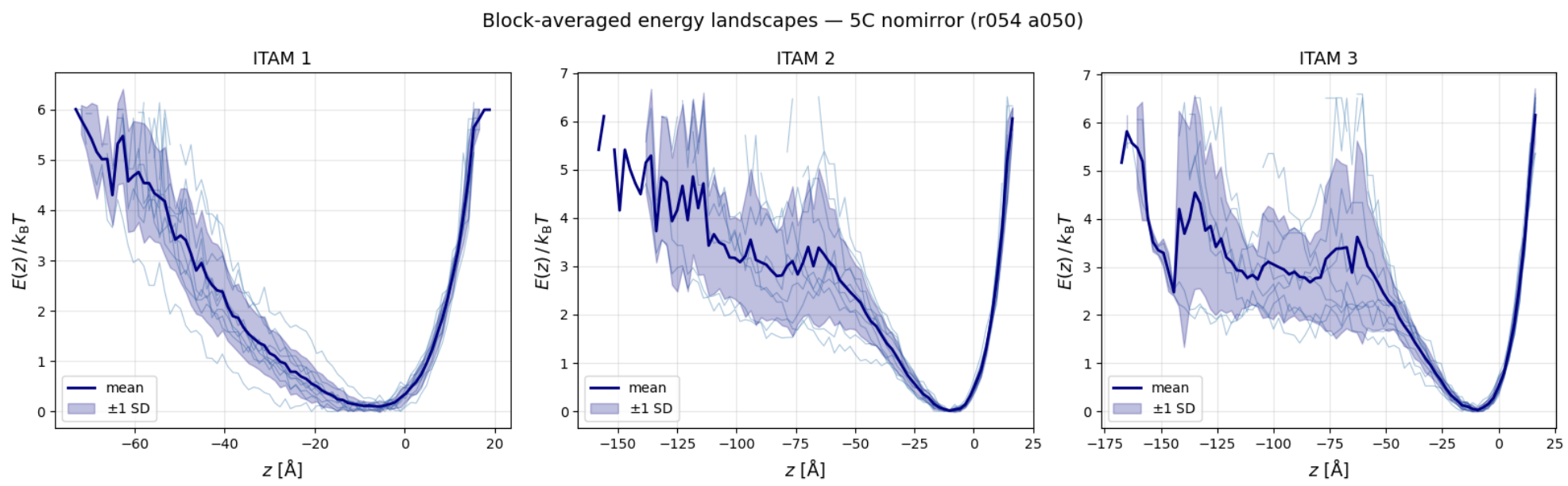

Fig. S4 Block-averaged energy landscapes for the 5-complex grid like (5C) configuration. Potential of mean force, $E(z)/k_{\mathrm{B}}T$, as a function of the reaction coordinate z for the three ITAM motifs (ITAM 1–3). Simulation data were divided into 10 blocks, and the energy landscape of each block is shown as a thin light-blue line. The dark-blue curve denotes the mean across blocks, and the shaded band indicates ±1 standard deviation

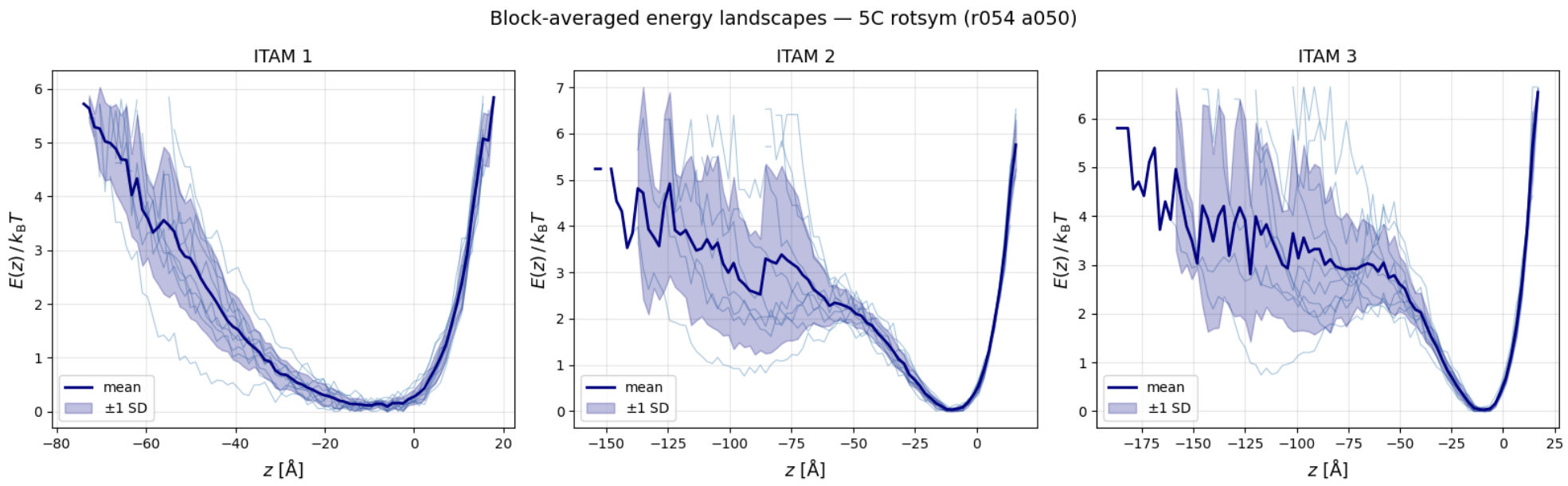

Fig. S5 Block-averaged energy landscapes for the 5-complex rotationally symmetric (5C rotsym) configuration. Potential of mean force, $E(z)/k_{\mathrm{B}}T$, as a function of the reaction coordinate z for the three ITAM motifs (ITAM 1–3). Simulation data were divided into 10 blocks, and the energy landscape of each block is shown as a thin light-blue line. The dark-blue curve denotes the mean across blocks, and the shaded band indicates ±1 standard deviation
